# Anaesthetics considerations for organ retrieval in the voluntary assisted dying patient – case report

**DOI:** 10.1186/s12871-026-03921-w

**Published:** 2026-05-21

**Authors:** Adam B Levin, Yashutosh Joshi, Nikunj Vaidhya, Joanne Kantianis, Peter S Macdonald

**Affiliations:** 1 Department of Anaesthesia and Pain Medicine, Barwon Health, 277-322 Ryrie St Geelong Victoria, Victoria, 3222 Australia; 2 Heart Transplant Unit, StVincent’s Hospital, Sydney, NSW Australia; 3 Victor Chang Cardiac Research Institute, Sydney, NSW Australia; 4 DonateLife Victoria, Barwon Health, Victoria, Australia

**Keywords:** Voluntary assisted dying, Euthanasia, Medical assisted dying, Anaesthesia, Organ donation, Case report

## Abstract

**Background:**

Voluntary assisted dying (VAD) is now legalised in several countries. Organ donation after VAD provides unique moral and logistical challenges. This report highlights the role of the anaesthetist in such cases.

**Case presentation:**

A 50-year-old man with end-stage amyotrophic lateral sclerosis underwent VAD and became an organ donor. Anaesthetic and logistical considerations during procurement are described. Advances in organ retrieval after VAD and psychosocial implications are briefly outlined.

**Conclusions:**

Organ donation after VAD presents unique opportunities and challenges. The anaesthetist plays a key role in ensuring both dignity for the patient and optimal conditions for organ retrieval.

## Background

Voluntary assisted dying (VAD) is now lawful in 17 countries worldwide [1]. The first successful kidney transplant in a human was conducted in 1950, and since then, transplant medicine has progressed to encompass a variety of organs with vastly improved survival rates [2].

Organ donation after VAD is a newer concept that started in Belgium in 2008 [3]. It is still practiced in only five countries worldwide: Australia, Belgium, Canada, the Netherlands and Spain [4]. In jurisdictions where the practise is more established VAD donated organs as a proportion of all donations is increasing and has been reported as high as 14% [5, 6]. Local guidelines for organ donation after VAD exist [7–9], but these guidelines do not provide instructions on the role of the anaesthetist.

VAD is legal in all states in Australia and the Australian Capital Territory. In Victoria, where this case occurred, two medical practitioners (either practitioner with fellowship with specialist medical college or vocationally trained general practitioner) who have undertaken mandatory legislative training are required to assess each patient for eligibility for VAD.

Organ donation is coordinated by DonateLife, whose specialised nursing staff guide families through the often daunting process. DonateLife and each jurisdiction’s VAD boards operate independently of each other. The VAD Boards in each Australian state are statutory oversight bodies responsible for monitoring compliance with assisted dying legislation, reviewing each case, and ensuring the process operates lawfully.

We present the case of a 50-year-old man with amyotrophic lateral sclerosis (ALS) who elected to undergo VAD and was an organ donor. The focus is on the role of the anaesthetist in the organ procurement process.

## Case presentation

Patient A was a 50-year-old male with end-stage ALS. Prior to his ALS diagnosis, two years earlier, he was fit and well with no medical comorbidities.

His main ALS symptoms were peripheral muscle weakness severely limiting function, requiring the use of a motorised wheelchair. The patient always expressed a desire not to have PEG feeds or noninvasive ventilation. As his disease progressed, he had an increasing inability to independently perform activities of daily living. A trial of riluzole (50 mg BD) did not significantly improve disease progression.

He undertook steps to arrange VAD according to the local legislation. This included assessment of decision-making capacity and ensuring that he was not coerced by two independent medical practitioners. He also expressed a desire to be an organ donor. Local protocols require VAD to be approved by the VAD Board prior to the person initiating the organ donation process.

One week before procurement, the patient underwent standard investigations, including ECHO, ECG, urine and sputum microscopy and culture, chest X-ray, serology, and blood and tissue typing in line with local guidelines [7].

On the day of donation, the patient was admitted to the local hospital intensive care facility. The patient was allowed to be accompanied by family and friends and was admitted to a single quarantined room within the ICU.

Prior to administering VAD medication, a team briefing was held with all members of the organ procurement team, including the VAD practitioner, organ donation co-ordinators, anaesthetist, visiting transplant surgeons, nursing staff and theatre technician. The VAD practitioner gave a briefing about the patient’s background and reasons for pursuing VAD and organ donation. Staff were given the opportunity to ask questions and reassured that participation in the case was not mandatory.

Logistical discussions included the process of communicating the administration of VAD medications to the retrieval team, documentation of death and transport to theatre, intubation, patient position, theatre setup, bronchoscopy, pulmonary recruitment and tracheal division.

The theatre was prepared to include appropriate equipment for procurement and storage of the organs and for intubating equipment. The surgeons and assistants scrubbed and gowned, the perfusion lines were primed, and the perfusate bags were cooled to prevent warming. Once prepared, the VAD coordinating practitioner was contacted to commence VAD drug administration. The anaesthesia and surgical staff were not involved in administration or VAD medications (performed by the VAD practitioner) or certification of death which was performed by ICU staff.

The patient was administered 25,000 IU of heparin before being administered VAD drugs in line with local guidelines. Because of advanced bulbar disease, the patient could not swallow and received intravenous medication administered by the coordinating VAD practitioner. He was monitored with a three-lead ECG and a pulse oximeter. The time of death was considered the loss of any electrical activity on the ECG. Blood pressure was not monitored.

Five minutes were allowed to elapse before certification of death by the VAD practitioner. Following the administration of VAD medications, the donor progressed to circulatory arrest (asystole) after nine minutes.

Following the five-minute stand-down period, the donor bed was immediately transferred to the operating theatre via a pre-planned route that minimised distance and potential exposure to other patients or staff not involved in the case. The transport was performed by hospital porters and the patient accompanied by the organ donation coordinator. The donor was intubated with an endotracheal tube and the cuff inflated to provide aspiration prophylaxis, but the patient was not yet ventilated. Given the proximity of the ICU to the operating theatre at the institution a decision was made to perform the intubation away from the family in the operating theatre.

The donor was then transferred to the operating table, prepped and draped to allow for the commencement of organ procurement via a direct procurement technique. ‘Knife to skin’ occurred at 14 min after the administration of VAD medications.

In this case cerebral circulation was not clamped prior to organ retrieval. The abdominal and thoracic organs were retrieved concomitantly, with an abdominal preservation flush given after 1.5 L of donor blood was drained from the right atrium to facilitate normothermic machine perfusion of the donor heart. The cardiothoracic retrieval team administered cardioplaegia and pneumoplaegia and lung protective ventilation with FiO2 of 50% was performed.

During dissection of the heart, bronchial alveolar lavage was performed by the anaesthetists for microbiological analysis. The surgeons and anaesthetists maintained close communication regarding lung inflation and tracheal stapling.

Given that it was donated after circulatory death (DCD), no anaesthetic drugs, monitoring or interventions other than those described above were necessary. The patient’s heart, lungs, kidneys and liver were successfully procured and prepared for donation as coordinated by the organ donation team. The asystolic warm ischaemic times for the heart and lungs were 11 min and 13 min, respectively. The organs were subsequently transported to other centres where they were successfully transplanted to recipients.

Post retrieval debriefing was undertaken by the organ donation coordinators. It was followed up a number of weeks later with an email to thank team members for their participation, give an update on the outcome of the donated organs and offer additional support.

## Discussion

Organ donation after VAD provides a unique opportunity for first-person consent for organ donation. It also allows for antemortem investigations and therapy, such as angiograms and IV heparin administration, which may lead to improved recipient outcomes.

It poses a moral quandary, including perceptions that life may be ended to obtain organs, conflicts of interest, increased burden on families who may oppose VAD, and potential loss of public trust if inadequately governed.

There is also a moral imperative to increase the pool of organs for donation. As well there is an ethical need to respect the autonomy and dignity of all patients, including VAD recipients, to donate their organs if they are eligible for transplantation. The case has been made for ethical directed organ donation after VAD, however, there are few reported instances of this happening [10–12].

Respecting donor autonomy in paramount in the process. The patient can self refer to the organ donation service or this may be done by the patient’s family member or VAD practitioner at the patient’s request. Once the request is received a face-to-face meeting is arranged by the organ donation coordinator to provide written and verbal information about the donation process.

To ensure the request is enduring, consent for organ donation can only be obtained at a subsequent meeting, usually two weeks later. A comprehensive guide is provided for the donation coordinator that includes a referral to the anaesthetist in charge at the hospital where the retrieval will occur, usually two days prior to organ procurement [7].

The anaesthetist plays a key role in the organ procurement process from a technical perspective, as a theatre coordinator and can also help staff to understand the donation process and the associated moral challenges.

Because lethal doses of medication are administered to facilitate VAD, there has been concern about potential effects on donated organs. Currently, no specific protocols exist to minimise end organ exposure, but limited case series have shown that short- and long-term outcomes of organs from VAD donors are comparable to, or better than, those from DCD donors who did not undergo VAD [13–15].

Due to the sensitive nature, the exact medications used for VAD are not published by the Victorian government but are similar to those used in other jurisdictions [16]. The medications are administered by the co-ordinating VAD practitioner.

In Australia, depending on local policy, some DCD withdrawals occur in the anaesthetic bay or on the operating table. These are logistics that need to be considered in VAD cases as well. In this case, the VAD process occurred in the ICU on a bed and not on an operating table. While it is advocated by retrieval teams for non-VAD DCD withdrawals to occur on an operating table in the anaesthetic bay, the VAD setup should be aimed at maximising donor comfort and dignity. With a well-briefed team, the transfer to the operating table can be smooth, with this experience demonstrating how VAD can achieve short warm ischaemic times.

While not yet available in Australia, the Netherlands has a system for delivery of sedation at home prior to induction of anaesthesia and transport to the hospital for VAD administration and organ retrieval [17]. In Canada a case of lung donation post VAD at home is also described [17, 18].

Given the time-critical nature, the VAD navigator, coordinating doctor and coordinating donation nurse briefed the family on the expected sequence of events, the need for expediency, and acknowledged that they would have little time with the deceased. The abrupt nature of this donation pathway requires a sensitive, understanding approach. In this context, the VAD navigator plays a central coordinating and supportive role. In Victoria, VAD navigators play a critical role in coordinating VAD and supporting patients and their loved ones through this complex and emotionally challenging experience. The navigators are typically nurses and are often the first point of contact for patients enquiring about VAD. They work in local health networks and help to link patients with VAD-trained clinicians.

Rapid securing of the airway is key for aspiration prophylaxis. All movements of the body increase the risk of aspiration and contamination of the lungs [19]. The time elapsed between medication administration and arriving at the operating suite rendered the facial tissue more rigid than that of a usual paralysed patient. As such, the use of the adjuncts allowed for rapid intubation. In our case, ‘Heart in a Box’ [20] technology was used to preserve the organ prior to donation [19]. Once the endotracheal tube is removed, the anaesthetist has no further formal role in monitoring or providing interventions. Instead, they should be available to assist with supporting theatre staff and running the theatre.

While meaningful and rewarding, being involved in organ procurement can also be a highly stressful and confronting experience for theatre staff. This is especially so in the case of organ donation after VAD where the planned nature of death, the need to maintain strict separation between VAD decision-making and donation processes, and the emotional impact of participating in both end-of-life care and organ retrieval contribute to ethical tension and psychological stress in a time-critical environment.

Institutions should have protocols available for debriefing after significant cases to ensure that all team members have a good understanding of the order of events and rationale for what has occurred. Ongoing support should also be available for all staff involved to ensure a safe and supportive work environment.

Consideration must also be given to staff belief systems when assigning theatre staff for VAD organ procurement. Conscientious objection to VAD remains common amongst health care workers [21] and guidelines for both medical and non medical health care workers have been [22, 23]. These emphasize a need for the practitioner with an objection to empathetically communicate their objection to either the employer or the patient.

Staff members who may normally be available to assist with organ procurement may have objections to being involved with VAD cases and should be allowed to opt out if they wish.

## Conclusions

Organ donation after VAD is increasingly relevant. This case highlights the anaesthetist’s pivotal role in minimising warm ischaemic time, ensuring safe airway management, and supporting the surgical team. Clear protocols, communication, and staff support are essential to optimise outcomes and respect donor dignity.

## Patient and carer perspectives

The case was discussed with the partner of the deceased, who expressed that for the patient and their family, organ donation was “the easiest part”. Knowing that there was benefit to others was a “privilege” and a “no brainer” for the family.

The partner also expressed that undergoing VAD in the hospital setting with a set date and time, rather than in the home setting, was of comfort to them.

## Data Availability

Not applicable. No datasets were generated or analysed for this case report.

## References

[1] White BP. Research Handbook on Voluntary Assisted Dying Law, Regulation and Practice. 1st ed. Cheltenham, UK: Edward Elgar Publishing; 2025.

[2] Nordham KD, Ninokawa S. The history of organ transplantation. Proc (Bayl Univ Med Cent). 2022;35(1):124–128. 10.1080/08998280.2021.1985889 PMC8682823 34970061

[3] Detry O, Laureys S, Faymonville M, De Roover A, Squifflet J, Lamy M, Meurisse M. Organ donation after physician-assisted death. Transpl Int. 2008;21(9):915. 18537924 10.1111/j.1432-2277.2008.00701.x

[4] Bollen J, Hempton C, Bhatia N, Tibballs J. Feasibility of organ donation following voluntary assisted dying in Australia: lessons from international practice. Med J Aust. 2023;219(5):202–5. 37393567 10.5694/mja2.52016

[5] Stukalin I, Olaiya OR, Naik V, Wiebe E, Kekewich M, Kelly M, Wilding L, Halko R, Oczkowski S. Medications and dosages used in medical assistance in dying: a cross-sectional study. CMAJ open. 2022;10(1):E19–26. 35042691 10.9778/cmajo.20200268 PMC8920593

[6] Banken-Vandewall A, Verberght H, Jansen N, De Jongh W, Van Dijk N, Van Mook W. Organ donation after euthanasia in the Netherlands, a summary of 12 years’ experience. Transplantation. 2025;109(12S):S60–1.

[7] Donate Life Victoria. Organ and Tissue Donation after Voluntary Assisted Dying. Donation Guideline; 2024.

[8] Downar J, Shemie SD, Gillrie C, Fortin M, Appleby A, Buchman DZ, Shoesmith C, Goldberg A, Gruben V, Lalani J, Ysebaert D, Wilson L, Sharpe MD. for Canadian Blood Services, the Canadian Critical Care Society, the Canadian Society of Transplantation and the Canadian Association of Critical Care Nurses: Deceased organ and tissue donation after medical assistance in dying and other conscious and competent donors: guidance for policy. CMAJ. 2019;191(22):E604–13. 31160497 10.1503/cmaj.181648 PMC6546574

[9] Bollen J, de Jongh W, Hagenaars J, van Dijk G, ten Hoopen R, Ysebaert D, Ijzermans J, van Heurn E, van Mook W. Organ Donation After Euthanasia: A Dutch Practical Manual. Am J Transplant. 2016;16(7):1967–72. 26842128 10.1111/ajt.13746

[10] van Dijk N, Shaw D, Shemie S, Wiebe K, van Mook W, Bollen J. Directed Organ Donation After Euthanasia. Transpl Int. 2023;36:11259. 10.3389/ti.2023.11259 PMC10262997 37324219

[11] Casey GM, Kekewich M, Naik VN, Hartwick M, Healey A. A request for directed organ donation in medical assistance in dying (MAID). Can J Anesth/J Can Anesth. 2020;67(7):806–9. 10.1007/s12630-020-01632-5 32207087

[12] Middleton C. Directed organ donation after medical assistance in dying: little to gain and much to lose. Can J Anesth/J Can Anesth. 2020;67(9):1310–1. 10.1007/s12630-020-01679-4 32356165

[13] Silva e Silva V, Silva A, Rochon A, Lotherington K, Hornby L, Wind T, Bollen J, Wilson LC, Sarti AJ, Dhanani S. Outcomes from organ donation following medical assistance in dying: A scoping review. Transpl Rev. 2023;37(1):100748. 10.1016/j.trre.2023.100748 36774782

[14] Slagter JS, Kimenai HJAN, van de Wetering J, et al. Kidney Transplant Outcome Following Donation After Euthanasia. JAMA Surg. 2024;159(12):1347–53. 39320864 10.1001/jamasurg.2024.3913 PMC11425192

[15] Slagter JS, Cappelle ML, Polak WG, den Hoed CM, van de Wetering J, Hellemons ME, Manintveld OC, van der Kaaij NP, Dor FJMF, Pengel LHM, Porte RJ, Minnee RC. Clinical outcomes of organ transplantation following donation after medical assistance in dying: a systematic review and meta-analysis. Transplantation. 2025;110(4):e876–e890. 10.1097/TP.0000000000005590 41385713

[16] Igor Stukalin OR, Olaiya V, Naik E, Wiebe M, Kekewich M, Kelly L, Wilding. Roxanne Halko, and, Simon Oczkowski: Medications and dosages used in medical assistance in dying: a cross-sectional study. CMAJ Open. 2022;10(1):E19–26. 35042691 10.9778/cmajo.20200268 PMC8920593

[17] Mulder J, Sonneveld JPC. Organ donation after medical assistance in dying at home. Can Med Association J (CMAJ). 2018;190(44):E1305–6. 10.1503/cmaj.170517 PMC6217602 30397157

[18] Healey A, Cypel M, Pyle H, Mills C, Heffren J, Katz D, Smith J, Teranishi R. Susan Lavery, Janice Beitel, Janet MacLean, Dwight Prodger, Shaf Keshavjee, Jonathan C. Yeung: Lung donation after medical assistance in dying at home. Am J Transplant. 2021;21(1):415–8. 32803817 10.1111/ajt.16267

[19] Saxena P, Zimmet AD, Snell G, Levvey B, Marasco SF, McGiffin DC. Procurement of lungs for transplantation following donation after circulatory death: the Alfred technique. J Surg Res. 2014;192(2):642–6. 25217512 10.1016/j.jss.2014.07.063

[20] Dhital KK, Iyer A, Connellan M, Chew HC, Gao L, Doyle A, Hicks M, Kumarasinghe G, Soto C, Dinale A, Cartwright B, Nair P, Granger E, Jansz P, Jabbour A, Kotlyar E, Keogh A, Hayward C, Graham R, Spratt P, Macdonald P. Adult heart transplantation with distant procurement and ex-vivo preservation of donor hearts after circulatory death: a case series. Lancet. 2015;385(9987):2585–91. 25888085 10.1016/S0140-6736(15)60038-1

[21] Sifris R. Voluntary assisted dying and conscientious objection: An analysis from Victoria, Australia. J Law Med. 2024;31(3):539–54. 39789683

[22] Lewis-Newby M, Wicclair M, Pope T, Rushton C, Curlin F, Diekema D, Durrer D, Ehlenbach W, Gibson-Scipio W, Glavan B, Langer RL, Manthous C, Rose C, Scardella A, Shanawani H, Siegel MD, Halpern SD, Truog RD. White DB: An Official American Thoracic Society Policy Statement: Managing Conscientious Objections in Intensive Care Medicine. Am J Respir Crit Care Med. 2015;191(2):219–27. 25590155 10.1164/rccm.201410-1916ST

[23] Isaac S, McLachlan A, Chaar B. Developing a framework for managing conscientious objection in healthcare - Australia. Health Policy Technol. 2025;14(2):100978.

